# Semantic Guidance Fusion Network for Cross-Modal Semantic Segmentation

**DOI:** 10.3390/s24082473

**Published:** 2024-04-12

**Authors:** Pan Zhang, Ming Chen, Meng Gao

**Affiliations:** College of Information, Shanghai Ocean University, No. 999 Hucheng Ring Road, Shanghai 201306, China; m210911552@st.shou.edu.cn (P.Z.); m210911521@st.shou.edu.cn (M.G.)

**Keywords:** semantic segmentation, cross-modal interactions, semantic guidance module

## Abstract

Leveraging data from various modalities to enhance multimodal segmentation tasks is a well-regarded approach. Recently, efforts have been made to incorporate an array of modalities, including depth and thermal imaging. Nevertheless, the effective amalgamation of cross-modal interactions remains a challenge, given the unique traits each modality presents. In our current research, we introduce the semantic guidance fusion network (SGFN), which is an innovative cross-modal fusion network adept at integrating a diverse set of modalities. Particularly, the SGFN features a semantic guidance module (SGM) engineered to boost bi-modal feature extraction. It encompasses a learnable semantic guidance convolution (SGC) designed to merge intensity and gradient data from disparate modalities. Comprehensive experiments carried out on the NYU Depth V2, SUN-RGBD, Cityscapes, MFNet, and ZJU datasets underscore both the superior performance and generalization ability of the SGFN compared to the current leading models. Moreover, when tested on the DELIVER dataset, the efficiency of our bi-modal SGFN displayed a mIoU that is comparable to the hitherto leading model, CMNEXT.

## 1. Introduction

Semantic segmentation presents a formidable challenge in several sectors, including autonomous driving, robotics, and virtual reality [1,2,3,4]. While neural networks have brought about a significant improvement in the accuracy and speed of RGB-based semantic segmentation in recent years, these models fall short when tasked with comprehensive 3D scene understanding. This is largely due to their inability to perceive crucial three-dimensional geometric information [5], thereby narrowing their practical applicability. Researchers have sought to mitigate these deficiencies by incorporating depth information [6] or thermal maps [7] into the semantic segmentation process. With advancements in sensor technology, an increasing number of sensors can now provide complementary detail to RGB images. However, the integration of multiple modalities is more challenging than a purely RGB-focused approach, as it requires effectually incorporating and leveraging the disparate characteristics of each modality. Therein lies the need for a flexible and adaptable network architecture specifically suited to multimodal segmentation.

**Figure 1:**
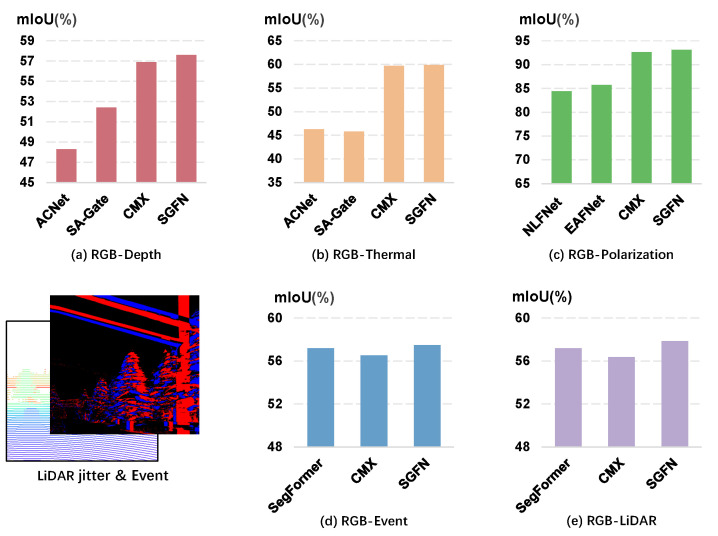
Comparative results of our SGFN on different benchmarks. (**a**) RGB and Depth on NYU Depth V2 [15] dataset; (**b**) RGB and Thermal on the MFNet [7] dataset; (**c**) RGB and degree of linear Polarization (DoLP) on ZJU [16] dataset; (**d**,**e**) RGB-Event and RGB-LiDAR on DELIVER [17] dataset.

The majority of current techniques for multimodal fusion can be categorized into two main strategies: early fusion and intermediate fusion. Early fusion involves merging RGB data with another modality at the channel level during the input stage [8,9,10]. Its downside is that it treats different modalities as uniform, thereby limiting its ability to fully exploit complementary information and pose challenges when applied to various modality combinations. On the flip side, intermediate fusion approaches [11,12,13] typically consist of parallel branches, where each branch is tasked with processing data from a specific modality. The features extracted at each layer from different modalities are then combined and passed through subsequent layers for semantic prediction. This layout is easily extendable to different RGB-based combinations. However, most existing models are tailor-made for specific combinations such as ACNet [14] and SA-Gate [11] for RGB-Depth data, resulting in underperformance when used for RGB-T segmentation (Figure 1). Therein lies the need for a robust and versatile multimodal network, one capable of adapting to diverse sensors without the necessity for dedicated architectures per modality. Unfortunately, the pursuit of this type of multimodal segmentation remains insufficient.

Moreover, many existing models [6,18,19] operate by assuming that each modality in multimodal data is invariably accurate. However, real-world measurement systems are prone to sensor faults, resulting in partial inaccuracies. For instance, issues such as LiDAR Jitter can introduce misalignments to sensing data [17]. Crucially, attempts at fusing such misaligned information could negatively impact overall segmentation performance, as depicted in Figure 1. Furthermore, multimodal data often display substantial levels of noise across the various sensing modalities. This noise can arise from various factors, including limited depth detection ranges [11], resulting in low-quality distance estimations and uncertainties revolving around dynamic cases [20]. It is critical to address these noise factors when dealing with multimodal data to ensure the reliability and accuracy of segmentation.

In light of these challenges, we present the semantic guidance fusion network (SGFN), a versatile cross-modal fusion network for multimodal semantic segmentation. Our model comprises two parallel transformers [21] paired with a novel Semantic Guidance Feature Fusion Module (SG-FFM) for pixel prediction. During the fusion, we introduce a semantic guidance module (SGM) for the extraction of supplementary multimodal information, which encompasses a learnable semantic guidance convolution (SGC). The SGC calculates the semantic distance of the supplementary modality to rectify the RGB data. This adjustment allows the fused feature to concentrate more on the complementary information while minimizing the negative impact of misalignments and noise from diverse modalities effectively.

In order to evaluate the efficacy of our proposed SGFN, we conducted exhaustive evaluations on six datasets, covering five distinct combinations of multiple modalities: RGB-Depth, RGB-Thermal, RGB-Polarization, RGB-Event, and RGB-LiDAR. Our results are noteworthy, with the highest mIoU of 57.6% on the NYU Depth V2 (RGB-D) dataset [15], 59.9% on the MFNet dataset (RGB-T) [7], and 93.1% on the ZJU dataset (RGB-P) [16]. Moreover, when tested on the DELIVER dataset [17], the performance of the SGFN is found to be on par with the previous best method. It is important to underline the fact that the SGFN surpasses specific architectures and outperforms existing multimodal methods, thereby affirming its effectiveness and superiority in delivering precise and robust multimodal semantic segmentation.

In summary, our research makes the following contributions:

1. We introduce a novel semantic guidance convolution (SGC) operation that calculates the similarity of adjacent pixels under the guidance of another modality to enhance the complementary cues and reduce the noise;
2. We further propose a new general multimodal segmentation network named SGFN, which is built on the SDC. This network is adept at effectively integrating and fusing bi-modal features from any amalgamation of modalities;
3. With comprehensive experiments on six datasets, our SGFN achieves state-of-the-art performance, covering RGB-D, RGB-T, RGB-P, RGB-E, and RGB-L tasks.

## 2. Related Works

### 2.1. Semantic Segmentation

Semantic segmentation is an intensive prediction task that needs to make predictions at the pixel level. Fully convolutional networks (FCNs) [22] first use a fully convolutional architecture to make pixel-wise prediction, which opens a new era of semantic segmentation. However, one upsampling step is insufficient to fully exploit the rich semantic information contained within the feature map. U-Net [23] uses an encoder-decoder structure to restore the image to its original resolution by multi-step upsampling while combining high-level features with low-level features through skip connections to improve accuracy. After that, a slew of advancements have been made to increase the receptive field for superior contextual comprehension. For example, PSPNet [24] introduced a pyramid pooling module to obtain contextual information at different scales. In parallel, the deepLab series [25,26,27,28] proposed atrous convolution, which broadened the receptive field without computational overhead. Some methods focus on improving semantic segmentation by enhancing boundaries [29,30,31] or applying attention blocks [32,33,34].

Recently, vision transformers [35] have been adopted as the backbone in dense prediction tasks [36,37] as well as in semantic segmentation tasks [21,38,39,40], demonstrating the effectiveness of global receptive fields. Although previous approaches have achieved impressive performance, they primarily rely on RGB images and, consequently, may suffer in challenging real-world scenarios. For instance, situations such as low-light conditions or fast-changing areas may expose the limitations of RGB images in accurately capturing minute scene details.

### 2.2. Multimodal Semantic Segmentation

Multimodal semantic segmentation is considerably enriched by the incorporation of disparate modalities, affording a more comprehensive understanding of scenes and bolstering overall performance. Numerous pieces of research have been undertaken on the fusion of RGB data with depth [6,9,11,19] and thermal [41,42,43,44] data, as they provide complementary information for scene analysis. Additional cues, such as polarization [45,46] and events [47,48], have also proven valuable in refining scene understanding. In the realms of autonomous driving, the integration of LiDAR data [49,50] and optical flow data [51] has drawn significant attention due to their essential contribution to perception tasks.

Despite these strides, many current approaches within this field tend to be tailored to specific modalities. This lack of adaptability to different combinations of sensing data impedes their broad application across various scenarios. CMX [52] offers a step towards handling this by putting forth a unified cross-modal fusion architecture for RGB-X segmentation, integrating cross-modal feature rectification and cross-attention feature fusion. Nonetheless, these methods often struggle when confronted with misaligned data resulting from sensor discrepancies.

By considering these constraints, we present a versatile framework that integrates cross-modal feature guidance to achieve robust segmentation. Our framework not only handles diverse combinations of modalities but also successfully mitigates the issues associated with misaligned data caused by sensor faults.

### 2.3. Central Difference Convolution

In order to improve edge performance, researchers have integrated gradient operators into vanilla convolutions, as the original convolution operation tends to smooth local features, resulting in decreased edge sharpness. By utilizing the fixed binary values, which are treated as filters, in convolution instead of learnable kernel weights, **local binary convolution (LBC)** [53,54] has been explored as an efficient alternative to traditional convolutions in various computer vision tasks. In the context of **central difference convolution (CDC)** [55,56,57], learnable kernels are employed to capture edge and texture details from the central difference map effectively; that is, $y_{c}=\sum_{0}^{n-1}w_{i}\cdot \left(x_{i}-x_{center}\right)$, where *w* indicates the kernel weights, and $x_{i}$ represents the surrounding pixel of the center entry in the local patch. It calculates the difference between pixel values in the horizontal and vertical directions to estimate the gradient information. By aggregating the gradient-level details within the local patch, CDC demonstrated impressive performance in anti-spoofing tasks. Furthermore, **pixel difference convolution (PDC)** [58] offers a more versatile approach to encoding local differences by modifying sampling strategies within the local region. This flexibility allows PDC to explore microstructures with greater adaptability. **Semantic difference convolution (SDC)** [59], on the other hand, draws inspiration from the diffusion process [60] and amplifies semantic boundary awareness by incorporating a similarity map, which is generated by calculating semantic similarity. However, all these aforementioned operators entirely concentrate on the extraction of features from the current modality to enhance edge representation. In contrast to previous work [11,52,61], we are dedicated to developing an innovative and effective operator-level solution that incorporates a guidance map derived from another modality to extract complementary information.

## 3. Proposed Method

In this section, we will initially provide a detailed elaboration of the SGFN framework designed for multimodal semantic segmentation (Section 3.1), then the Semantic Guidance Feature Fusion Module (SG-FFM) is covered in Section 3.2, the Semantic Guidance Convolution (SGC) is covered in Section 3.2.1, and the corresponding semantic guidance module (SGM) is covered in Section 3.2.2.

### 3.1. Framework Overview

**Figure 2:**
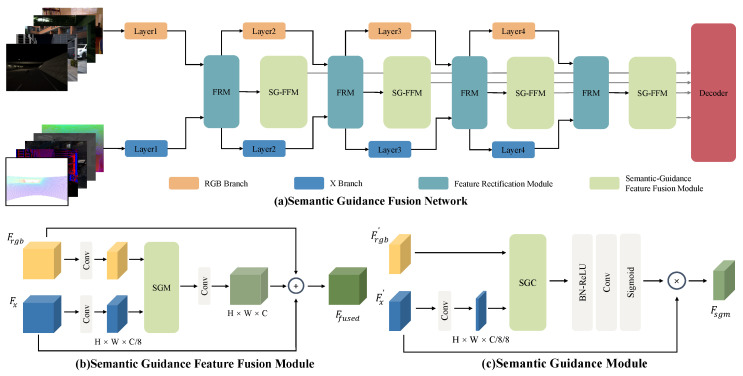
(**a**) The overall architecture of our SGFN. It is composed of a parallel encoder to fuse RGB data and other modality data. (**b**) Details of the Semantic Guidance Feature Fusion Module (SG-FFM). (**c**) Detailed architecture of semantic guidance module (SGM).

In Figure 2a, We apply an encoder-decoder structure to our SGFN. The encoder consists of two parallel backbones designed to extract features from RGB images and other modalities, including Depth, Thermal, Polarization, Event, LiDAR, and more. By following most of the previous works [24,32,62], we used a four-stage structure in the backbone to extract pyramidal features from each modality. At the end of each stage, the features from different modalities are rectified by the cross-modal feature rectification module (FRM) [52], which is crucial to promote interactions and reduce noise. Thus, the calibrated features are sent back to the backbone to continue the extraction of deeper characteristics. Moreover, as shown in Figure 2b, we designed a Semantic Guidance Feature Fusion Module (SG-FFM) to fuse the rectified features at each stage of the encoder, termed *F*. Within SGM, we introduce a semantic guidance convolution operator that takes the feature maps of another modality as a guide, thereby formulating a more enriched feature map. Ultimately, the features of four stages, $F\in \left\{F_{1},F_{2},F_{3},F_{4}\right\}$, are passed to the decoder to predict the semantic map.

### 3.2. Semantic Guidance Feature Fusion Module

It is worth highlighting that the pixels share the same semantic label, demonstrating a higher degree of similarity. Extracting valuable features is essential when dealing with different modalities, as they often possess complementary information [11,14]. In this subsection, shown in Figure 2, we propose a new learnable approach known as the Semantic Guidance Feature Fusion Module (SG-FFM) to interact with two distinct modalities effectively. The SG-FFM consists of a semantic guidance module, comprising a parametric semantic guidance convolution operator, followed by a straightforward feature fusion process that generates enhanced features. Specifically, as indicated in Figure 2b, the channel dimensions of both modalities are initially compressed by a factor of one-eighth for reduction. Next, the modalities are sent into the SGM, which generates enhanced features. Subsequently, the outputs of SGM are convolved to achieve channel-wise alignment. At last, the generated outputs are integrated with “X” features and RGB features by performing an element-wise summation.

#### 3.2.1. Semantic Guidance Convolution

**Figure 3:**
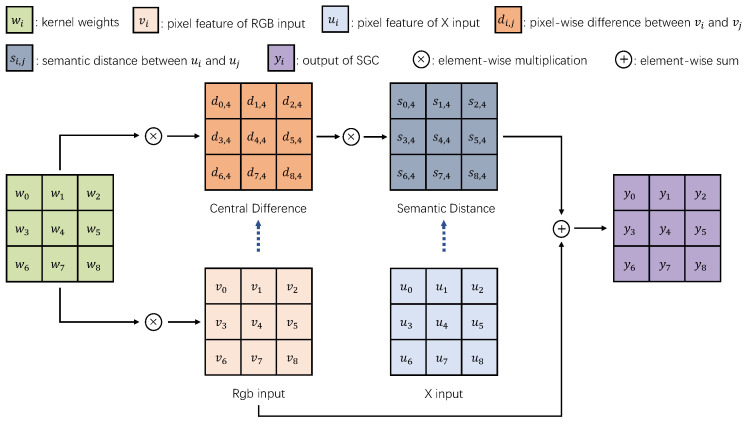
Semantic guidance convolution.

By taking inspiration from SDC, which effectively applies semantic difference convolution to mimic the diffusion process and has shown substantial improvements in boundary performance, our approach also incorporates central difference into our SGC, emulating the diffusion process. The SGC encompasses two primary stages. In our method, the sampling step over the input feature follows a similar pattern to vanilla convolution. However, an amendment is introduced in the succeeding amalgamation step. The semantic guidance convolution, as depicted in Figure 3, focuses on aggregating the center-oriented gradient of local patches from complementary modalities. SGC generates the output value *Y* by taking the feature map *V* and another modality’s guidance map, *U*, as input. Consequently, we express the formula as follows:

(1) $$Y_{c}=\sum_{i=0}^{n-1}W_{i}\cdot S\left(U_{i}-U_{center}\right)\cdot \left(V_{i}-V_{center}\right)$$

where *i* enumerates the pixels in the current patch. The first term, *W*, represents the learnable kernel weights, which have the same size as *U* and *V*. The second term, $S\left(U_{i}-U_{center}\right)$, known as the semantic guidance term, quantifies the semantic distance between the central pixel and its surrounding counterparts at the same location in another modality. Specifically, $S\left(U_{i}-U_{center}\right)={∥U_{i}-U_{center}∥}_{2}$. The last term $\left(V_{i}-V_{center}\right)$, known as the central difference term, evaluates the disparity between adjacent pixels at the pixel level. The semantic guidance term (S) and central difference term (D) have a mutual influence on each other, with S capable of acting as a guiding factor to enhance or suppress D, and conversely, D can also have a similar effect on S. This dynamic interaction enables our network to successfully extract complementary information and effectively handle challenges such as noise or misalignments.

In semantic segmentation tasks, assimilating information from both the intensity and gradient levels holds critical value. Therefore, by combining vanilla convolution with semantic guidance convolution, we augment the capability to capture diverse and informative features, leading to improved robustness and accuracy in semantic segmentation. As a result, the semantic guidance convolution can be represented as

(2) $$\begin{array}{cc} Y_{c}= & \theta \cdot \underset{\mathrm{semantic}\;\mathrm{guidance}\;\mathrm{term}}{\underbrace{\sum_{i=0}^{n-1}W_{i}\cdot S\left(U_{i}-U_{center}\right)\cdot \left(V_{i}-V_{center}\right)}}\\ \; & +\left(1-\theta \right)\cdot \underset{\mathrm{vanilla}\;\mathrm{term}}{\underbrace{\sum_{i=0}^{n-1}W_{i}\cdot V_{i}}}\; \end{array}$$

In this context, $\theta \in \left[0,1\right]$ serves as a hyperparameter to govern the trade-off between the gradient term and intensity term. The ablation of θ will be demonstrated in Section 5.5.

#### 3.2.2. Semantic Guidance Module

As previously discussed, SGC concentrates on drawing out complementary information from different modalities. In order to further augment the cross-modal interactions, we introduce an efficient yet simple module called the **semantic guidance module (SGM)**, which builds upon our proposed SGC approach.

As shown in Figure 2c, our SGM utilizes a branching framework that accommodates two inputs,

(3) $$F^{sgm}=SGM\left(F^{rgb},F^{x}\right)$$

where $F^{rgb}\in R^{C\times H\times W}$ is the RGB features, and $F^{x}\in R^{C\times H\times W}$ is the guidance feature from another modality. The process can be formulated as

(4) $$\begin{array}{cc} \; & {\hat{F}}^{x}=Conv_{1\times 1}\left(F^{x}\right) \end{array}$$

(5) $$\begin{array}{cc} \; & F^{sgc}=\mathrm{BN-Relu}\left(SGC\left(F^{rgb},{\hat{F}}^{x}\right)\right) \end{array}$$

(6) $$\begin{array}{cc} \; & w=Sigmoid\left(Conv_{1\times 1}\left(F^{sgc}\right)\right) \end{array}$$

(7) $$\begin{array}{cc} \; & F^{sgm}=w\cdot F^{sgc} \end{array}$$

In Equation (5), by utilizing a 1×1 convolution, the feature $F^{x}$ is reduced to one-eighth (by default) of its original size along the channel dimension. Afterward, the bi-modal inputs are fed into the proposed SGC, which is then followed by batch normalization (BN) [63] and the ReLU [64] activation function. Finally, a Sigmoid function is applied to compute the attention weight after recovering the channel with a 1×1 convolution.

## 4. Experiments

### 4.1. Datasets

In order to validate our proposed SGFN (semantic guidance fusion network), we conducted experiments on three datasets relating to RGB-Depth semantic segmentation, as well as datasets involving combinations of the RGB-Thermal, RGB-Polarization, RGB-Event, and RGB-LiDAR modalities.

**NYU Depth V2** [15] is an indoor RGB-D dataset with a total of 1449 images categorized into 40 classes, displayed at a resolution of 640 × 480 pixels. The dataset is divided into a training set of 795 images and a testing set of 654 images.

**SUN-RGBD** [65] is an indoor RGB-D dataset containing 10,335 images classified into 37 categories. It’s split into 5285/5050 for training/testing. We cropped and resized the image to 480×480.

**Cityscapes** [3] is a benchmark for outdoor datasets featuring urban street scenes; it is divided into training/validation/testing sets of 2975/500/1525 samples, respectively. It comprises fine annotations for 19 different classes. We took an input at the resolution of 1024×512.

**RGB-T MFNet** [7]. The MFNet dataset comprises 1569 RGB-thermal pairs captured from urban street scenes, with a resolution of 640×480 and eight classes. Among these, 820 pairs were captured during the daytime, and the remaining were captured at night.

**RGB-P ZJU** [16] is an RGB-P dataset collected from college street scenes, which comprises a total of 394 images, with 344/50 pairs for training/evaluation. Each image pair in the dataset is annotated for eight distinct semantic classes. Each image was cropped and resized to 612×512.

**DELIVER** [17] consists of Depth, LiDAR, Event, and RGB data, each with a size of 1024×1024. It comprises a total of 7885 samples, including sensor faults such as LiDAR-Jitter and Event Low-resolution. It is divided into training (3983), validation (2005), and testing (1897) sets, with 25 classes. Our study focuses on exploring the combinations of the RGB-Event and RGB-LiDAR modalities.

### 4.2. Implementation Details

We used the parallel Mix-Transformer-B2 (MiT-B2) [21] architecture pre-trained on the ImageNet [66] dataset as the backbone and UPernet-decoder [67] for our model. We trained our models on Pytorch 1.8.1 with four 3090 GPUs. We chose the AdamW optimizer [68] with epsilon $10^{-8}$, and weight decay $10^{-2}$. The initial learning rate (LR) was set as $6\times 10^{-5}$ with a poly strategy. We warmed up the first 10 epochs with 0.1× the original LR. We used the cross-entropy loss function. Throughout the training, we performed data augmentation by random flipping and random scaling. For NYU Depth V2 and SUN RGB-D, we used multiscale flip testing for a fair comparison. Like most of the previous works [15,65,69], we applied two common metrics, pixel accuracy (Pixel Acc.) and mean intersection over union (mIoU), to evaluate our model.

## 5. Experiment Results and Analyses

In this section, we present extensive experiment results on six multimodal benchmarks to verify the efficacy of our proposed SGFN for multimodal semantic segmentation. The results are compared with state-of-the-art methods, as shown in the following subsections.

### 5.1. Results of the RGB-Depth Datasets

**NYU Depth V2:** The results of the NYU Depth V2 dataset with 40 categories are shown in Table 1. It is evident that the exceptional performance of our approach surpasses previous methods in terms of the scores. Specifically, our proposed method (utilizing MiT-B2) already achieves remarkable results with a mIoU of 53.4%. In building upon this success, our SGFN models based on MiT-B4 and B5 demonstrate significant enhancements, significantly elevating the mIoU to 56.9% and 57.6%, respectively. These outstanding improvements highlight the effectiveness and superiority of our approach in cross-modal semantic segmentation.

**SUN-RGBD:** As shown in Table 2, our method achieves the best scores in two evaluation metric when compared with previous works. Precisely, our models leveraging MiT-B2 and B5 achieve impressive mIoU scores of 50.4% and 52.8%, respectively. These results serve as strong evidence of the remarkable effectiveness of our proposed method and underscore the superiority of our approach in multimodal semantic segmentation.

**Cityscapes:** In order to examine its applicability to outdoor environments, we evaluated the performance of SGFN on the Cityscapes dataset. The findings, displayed in detail in Table 3, provide a comparative study between our models and the cutting-edge RGB and RGB-D methods. Remarkably, our RGB-D approach demonstrates a notable enhancement of 0.9% in mIoU over the MiT-B2 (RGB) model. Furthermore, our method leveraging MiT-B4 achieves a leading score of 83.1%, outperforming all other RGB-D techniques available in the field.

### 5.2. Results of the RGB-Thermal Dataset

In Table 4, the results of a set of experiments on the MFNet dataset to evaluate the generalization capability of our model can be seen. Our SGFN model, utilizing MiT-B4, achieves state-of-the-art performance, surpassing CMX [52] by 0.2% in mIoU. Our methods based on MiT-B2 already surpass RGB-only models as well as RGB-T methods such as FEANet [61], ABMDRNet [12], and GMNet [43], achieving a mIoU of over 59%. Models designed for RGB-D segmentation, such as ACNet [14] and SA-Gate [11], are not applicable to RGB-T scenarios, as they only interact in the channel dimension while neglecting the crucial pixel-wise information. It is evident that our SGFN achieves remarkable success in extracting multimodal information effectively.

As shown in Table 5, we conducted separate experiments for both daytime and nighttime conditions. In the daytime scenario, our method achieves a comparable mIoU of 52.5% compared to CMX [52]. However, in nighttime conditions, despite the noise caused by poor light, our model surpasses all other RGB-T methods, achieving an impressive mIoU that exceeds 60.0%.

### 5.3. Results of the RGB-Polarization Dataset

In order to demonstrate the generality of our method, we conducted further evaluations on the ZJU-RGB-P dataset [16]. By leveraging trichromatic representations, which have proven to be more informative than monochromatic representations [16,84], we introduced the trichromatic degree of linear polarization (DoLP) as supplementary data. In Table 6, our approach utilizing MiT-B2 already surpasses all other RGB-P models, achieving a mIoU of 92.8% and outperforming the previous leading model CMX by 0.2%. Furthermore, our top-performing model with MiT-B4 attains a leading score of 93.1%, validating the generalizability of our SGFN approach on RGB-P data.

### 5.4. Results of the DELIVER Dataset

In Table 7, we compare our SGFN with recent models on the DELIVER dataset to study the generalizability of our approach in RGB-Event and RGB-LiDAR. Overall, SGFN, with MiT-B2, obtains state-of-the-art status for fusing RGB data and Event data, reaching the same mIoU (57.48) as CMNeXt. Additionally, our model performs comparably to CMNeXt in the fusion of RGB and LiDAR data. These results show that our SGM plays a positive role in fusing dense-sparse data.

### 5.5. Ablation Study

In order to gain a deeper understanding of how the various components of our architecture contribute to the segmentation task, we conducted a comprehensive set of ablation studies. For a fair comparison, we took MiT-B2 as the backbone to evaluate the performance of our model on the NYU Depth V2 dataset.

**Ablation of the SGFN architecture.** As shown in Table 8, we performed ablation experiments on our SGFN architecture. If we remove the SGM module, the complementary modalities are simply combined with average fusion. The results show a decrease in mIoU by 1.5% compared to the baseline. This decline highlights the critical role of our SGM in facilitating effective cross-modal fusion. Similarly, when removing the FRM module, the modalities are extracted independently, without any interaction or influence on each other during the process. This leads to a decline in the value by 2.2%, indicating the importance of the FRM module in enabling robust cross-modal interactions between the RGB feature and the supplementary feature.

**Comparing SGC with other Convolutions**: In order to evaluate the effectiveness of semantic guidance convolution (SGC) in cross-modal fusion, we conducted experiments by replacing the SGC in SGM with two alternative convolutions: vanilla convolution [86] and CDC. The results, as shown in Table 8, demonstrate that SGC outperforms vanilla convolution by a margin of 1.0%. Interestingly, the CDC achieves a much lower score, which may be attributed to its design for edge detection rather than semantic segmentation tasks. These findings emphasize the irreplaceable role of our SGC in achieving accurate multimodal semantic segmentation.

**Figure 4:**
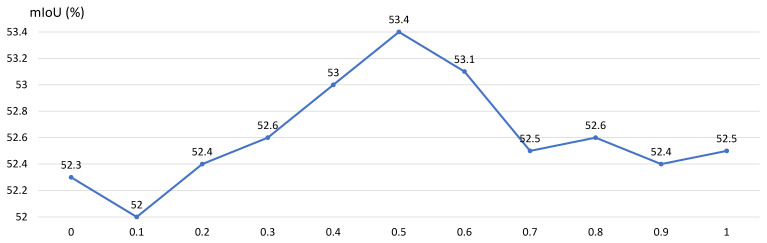
Impact of θ on SGC.

**Impact of** θ **in SGC**: As mentioned in Section 3.2.1, the parameter θ controls the influence of the gradient-level details and intensity-level details in SGC. We systematically varied θ as a hyperparameter in the range of 0 to 1 to investigate the impact of the semantic guidance term on the overall performance of SGC. Notably, in Figure 4, when θ exceeds 0.3, SGC consistently outperforms vanilla convolution (θ=0), indicating the superior performance of SGC in capturing both gradient-level and intensity-level details. Since the highest performance is observed when θ is set to 0.5, we set θ=0.5 as the default configuration for all experiments.

**Impact of Kercel Size in SGC**: In order to evaluate the impact of kernel size and dilation rate, we conducted an ablation study. As shown in Table 9, increasing the size of the kernel in SGC did not result in any improvement. This suggests that enlarging the receptive fields may not be necessary for our SGC, as it primarily focuses on capturing bi-modal interactions in the local region. Additionally, we observe negative effects when increasing the dilation rate, which potentially results in the loss of local details.

### 5.6. Qualitative Analysis

**Figure 5:**
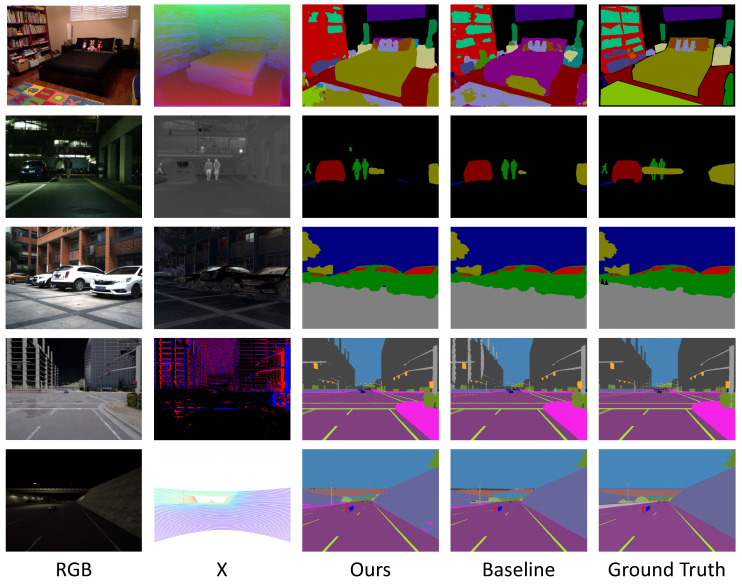
Visualization of qualitative comparison of RGB-only and our SGFN, arranged from top to bottom, showcasing the results for the RGB-Depth, RGB-Thermal, RGB-Polarization (AoLP), RGB-Event, and RGB-LiDAR semantic segmentation tasks.

**Visualization of Prediction Results**: Figure 5 displays the visual outcomes of our cross-modal segmentation, indicating the proficiency of our method across various modalities. Specifically, for RGB-D results, the SGM successfully discerns depth details and rightly segments the bed. Conversely, the baseline, which relies exclusively on RGB images, misclassifies the bed as a sofa, thereby demonstrating the enhanced accuracy of our approach. In the context of RGB-T segmentation, the baseline model struggles under low illumination conditions, leading to erroneous segmentations. In contrast, our network overcomes this challenge by effectively correcting the errors and achieving clearer distinctions between objects and persons. Moreover, the integration of polarization cues in RGB-P segmentation enables the more precise segmentation of car and glass areas, further enhancing the overall performance of our method. Notably, our SGM exhibits robust generalization capabilities in dynamic scenes, effectively accommodating moving objects and improving segmentation accuracy. By leveraging the advantages of LiDAR points, our network successfully segments the complete structure of the bridge, whereas the baseline misclassifies it as part of the sky. These qualitative analyses collectively demonstrate the strong generalization ability of our approach across multiple modalities.

**Figure 6:**
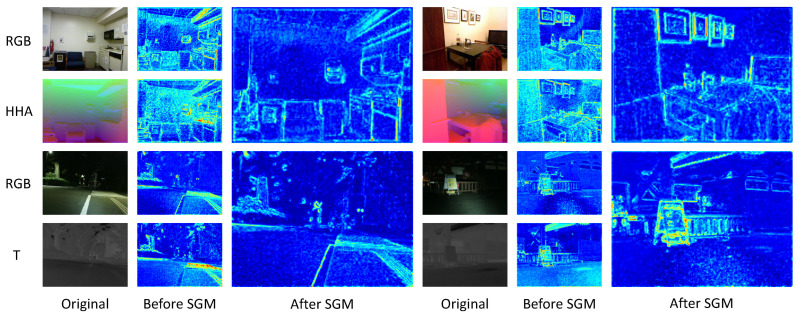
Visualization of the feature maps before and after the application of the SGM.

**Visualization of Feature Maps**: In order to demonstrate the efficacy of our semantic guidance module (SGM), we visualized the feature maps extracted from the first layer of the backbone. By comparing the feature maps before and after applying SGM, as depicted in Figure 6, we can clearly see the remarkable ability of our semantic guidance fusion network (SGFN) to accentuate edge details and effectively suppress noise for both RGB-D and RGB-T tasks. In RGB-D tasks, depth features provide crucial geometric information that can complement RGB features, enhancing edge performance. For RGB-T tasks, thermal images become more important, especially in nighttime scenarios where RGB images may struggle due to inadequate lighting conditions. The effective utilization of these complementary modalities demonstrates the efficacy of our SGM.

## 6. Conclusions

In this study, we introduce a novel approach for universal cross-modal semantic segmentation called semantic guidance fusion network (SGFN). Our method leverages a vision transformer architecture to extract meaningful features from diverse modalities. We put forward a cross-modal Semantic Guidance Feature Fusion Module (SG-FFM) for comprehensive interactions between diverse modalities. SG-FFM utilizes a novel operator-level operation to augment the RGB feature in boundary awareness so as to enhance the performance of multimodal fusion. Specifically, we calculate the semantic distance of the supplementary information to guide the pixel-wise relevance derived from RGB information. The extensive experiments conducted on six benchmark datasets, including RGB-Depth, RGB-Thermal, RGB-Polarization, RGB-Event, and RGB-LiDAR combinations, demonstrate the superior performance of our proposed SGFN compared to existing state-of-the-art methods for cross-modal semantic segmentation.

In the future, our goal is to tackle the challenge of cross-modal fusion beyond the current scope by adapting the SGFN framework to accommodate the integration of three or more distinct sensor data types.

## Figures and Tables

**Table 1:** Results of NYU Depth V2. “*” denotes the multi-scale test.

Method	mIoU (%)	Acc (%)
3DGNN [70]	43.1	-
ACNet [14]	48.3	-
PADNet [71]	50.2	62.3
PAP [72]	50.4	62.5
Swin-RGBD [73]	50.9	64.2
TransD [74]	55.5	69.4
SGNet [75]	51.1	76.8
ShapeConv [9]	51.3	76.4
SA-Gate [11]	52.4	77.9
CMX (MiT-B2) * [52]	54.4	79.9
CMX (MiT-B5) * [52]	56.9	80.1
SGFN (Mit-B2) *	53.4	78.5
SGFN (Mit-B5) *	57.6	80.5

**Table 2:** Results of SUN RGB-D. “*” denotes the multi-scale test.

Method	mIoU (%)	Acc (%)
3DGNN [70]	45.9	-
ACNet [14]	48.1	-
SGNet [75]	48.6	82.0
ShapeConv [9]	48.6	82.2
NANet [76]	48.8	82.3
PDCNet [6]	49.2	-
CANet [77]	49.3	60.5
TransD [74]	51.9	64.1
SA-Gate [11]	49.4	82.5
CMX (MiT-B2) * [52]	49.7	82.8
CMX (MiT-B5) * [52]	52.4	83.8
SGFN (Mit-B2) *	50.8	83.0
SGFN (Mit-B5) *	53.1	84.1

**Table 3:** Results of Cityscapes.

Method	Modal	Backbone	mIoU (%)
SwiftNet [78]	RGB	ResNet-18	70.4
ESANet [19]	RGB	ResNet-50	79.2
DANet [32]	RGB	ResNet-101	81.5
SegFormer [21]	RGB	MiT-B2	81.0
SegFormer [21]	RGB	MiT-B4	82.3
RFNet [79]	RGB-D	ResNet-18	72.5
PADNet [71]	RGB-D	ResNet-50	76.1
ESANet [19]	RGB-D	ResNet-50	80.0
SA-Gate [11]	RGB-D	ResNet-50	80.7
SA-Gate [11]	RGB-D	ResNet-101	81.7
CMX [52]	RGB-D	MiT-B2	81.6
CMX [52]	RGB-D	MiT-B4	82.6
SGFN	RGB-D	MiT-B2	81.6
SGFN	RGB-D	MiT-B4	83.0

**Table 4:** Per-class comparison on the MFNet dataset for RGB-Thermal semantic segmentation.

Method	Modal	Unlabeled	Car	Person	Bike	Curve	Car Stop	Guardrail	Color Cone	Bump	mIoU
DANet [32]	RGB	96.3	71.3	48.1	51.8	30.2	18.2	0.7	30.3	18.8	41.3
SegNet [75]	RGB	96.7	65.3	55.7	51.1	38.4	10.0	0.0	12.0	51.5	42.3
UNet [23]	RGB	96.9	66.2	60.5	46.2	41.6	17.9	1.8	30.6	44.2	45.1
PSPNet [24]	RGB	96.8	74.8	61.3	50.2	38.4	15.8	0.0	33.2	44.4	46.1
ERFNet [80]	RGB	96.7	67.1	56.2	34.3	30.6	9.4	0.0	0.1	30.5	36.1
DUC [81]	RGB	97.7	82.5	69.4	58.9	40.1	20.9	3.4	42.1	40.9	50.7
HRNet [82]	RGB	98.0	86.9	67.3	59.2	35.3	23.1	1.7	46.6	47.3	51.7
SegFormer-B2 [21]	RGB	97.9	87.4	62.8	63.2	31.7	25.6	9.8	50.9	49.6	53.2
SegFormer-B4 [21]	RGB	98.0	88.9	64.0	62.8	38.1	25.9	6.9	50.8	57.7	54.8
MFNet [7]	RGB-T	96.9	65.9	58.9	42.9	29.9	9.9	0.0	25.2	27.7	39.7
SA-Gate [11]	RGB-T	96.8	73.8	59.2	51.3	38.4	19.3	0.0	24.5	48.8	45.8
ACNet [14]	RGB-T	96.7	79.4	64.7	52.7	32.9	28.4	0.8	16.9	44.4	46.3
RTFNet [44]	RGB-T	98.5	87.4	70.3	62.7	45.3	29.8	0.0	29.1	55.7	53.2
AFNet [83]	RGB-T	98.0	86.0	67.4	62.0	43.0	28.9	4.6	44.9	56.6	54.6
ABMDRNet [12]	RGB-T	98.6	84.8	69.6	60.3	45.1	33.1	5.1	47.4	50.0	54.8
FEANet [61]	RGB-T	98.3	87.8	71.1	61.1	46.5	22.1	6.6	55.3	48.9	55.3
GMNet [43]	RGB-T	97.5	86.5	73.1	61.7	44.0	42.3	14.5	48.7	47.4	57.3
CMX (MiT-B2) [52]	RGB-T	98.3	89.4	74.8	64.7	47.3	30.1	8.1	52.4	59.4	58.2
CMX (MiT-B4) [52]	RGB-T	98.3	90.1	75.2	64.5	50.2	35.3	8.5	54.2	60.6	59.7
SGFN (MiT-B2)	RGB-T	98.3	89.4	76.0	66.1	49.3	32.7	10.9	52.4	56.1	59.0
SGFN (MiT-B4)	RGB-T	98.4	90.9	76.7	66.1	49.2	35.7	7.5	55.1	59.1	59.9

**Table 5:** Experiments on the nighttime and daytime images of the MFNet dataset.

Method	Modal	Daytime mIoU (%)	Nighttime mIoU (%)
SegFormer-B2 [21]	RGB	48.6	49.2
SegFormer-B4 [21]	RGB	49.4	52.4
GMNet [43]	RGB-T	49.0	57.7
MFNet [7]	RGB-T	36.1	36.8
RTFNet [44]	RGB-T	45.8	54.8
ABMDRNet [12]	RGB-T	46.7	55.5
CMX (MiT-B2) [52]	RGB-T	51.3	57.8
CMX (MiT-B4) [52]	RGB-T	52.5	59.4
SGFN (MiT-B2)	RGB-T	52.0	58.7
SGFN (MiT-B4)	RGB-T	52.5	60.0

**Table 6:** Results of the ZJU dataset for RGB-Polarization segmentation.

Method	Modal	mIoU (%)
SwiftNet [78]	RGB	80.3
SegFormer-B2 [21]	RGB	89.6
NLFNet [84]	RGB-P	84.4
EAFNet [16]	RGB-P	85.7
CMX (SegFormer-B2) [52]	RGB-AoLP	92.0
CMX (SegFormer-B4) [52]	RGB-AoLP	92.6
CMX (SegFormer-B2) [52]	RGB-DoLP	92.2
CMX (SegFormer-B4) [52]	RGB-DoLP	92.5
SGFN (SegFormer-B2)	RGB-DoLP	92.8
SGFN (SegFormer-B4)	RGB-DoLP	93.2

**Table 7:** Results of the DELIVER datasets for RGB-Event and RGB-LiDAR semantic segmentation.

Method	Modal	Backbone	mIoU (%)
HRFuser [85]	RGB	HRFormer-T	47.95
SegFormer [21]	RGB	MiT-B2	57.20
HRFuser [85]	RGB-Event	HRFormer-T	42.22
CMX [52]	RGB-Event	MiT-B2	56.52
CMNeXt [17]	RGB-Event	MiT-B2	57.48
SGFN	RGB-Event	MiT-B2	57.48
HRFuser [85]	RGB-LiDAR	HRFormer-T	43.13
CMX [52]	RGB-LiDAR	MiT-B2	56.37
CMNeXt [17]	RGB-LiDAR	MiT-B2	58.04
SGFN	RGB-LiDAR	MiT-B2	57.70

**Table 8:** Albation on the SGFN architecture.

Structure	mIoU (%)
SGFN (MiT-B2)	53.4
-without SGM	51.6 (−1.8)
-without FRM	50.6 (−2.8)
-with CDC instead of SGC	51.4 (−2.0)
-with vanilla instead of SGC	52.3 (−1.1)
-with SDC instead of SGC	52.6 (−0.8)

**Table 9:** Impact of the kernel size of the SGC operator.

Kernel Size	Dilation Rate	Pixel Acc. (%)	mIoU (%)
3 × 3	1	78.5	53.4
3 × 3	3	78.2	53.1
3 × 3	5	77.9	52.7
5 × 5	1	78.4	53.3
7 × 7	1	78.2	52.9

## Data Availability

The data presented in this study are available on request from the corresponding author.
